# Hearing impairment after asphyxia and neonatal encephalopathy: a Norwegian population-based study

**DOI:** 10.1007/s00431-023-05321-5

**Published:** 2023-11-22

**Authors:** Dagny Hemmingsen, Dag Moster, Bo Engdahl, Claus Klingenberg

**Affiliations:** 1 Department of Otorhinolaryngology and Head and Neck Surgery, University Hospital of North Norway, N-9038 Tromsø, Norway; 2 Paediatric Research Group, Faculty of Health Sciences, UiT-The Arctic University of Norway, Tromsø, Norway; 3 Department of Global Public Health and Primary Care, University of Bergen, Bergen, Norway; 4 Department of Pediatrics, Haukeland University Hospital, Bergen, Norway; 5 Department of Physical Health and Ageing, Norwegian Institute of Public Health, Oslo, Norway; 6 Department of Paediatrics and Adolescence Medicine, University Hospital of North Norway, Tromsø, Norway

**Keywords:** Apgar score, Neonatal morbidity, Hypoxic-ischemic encephalopathy, Hearing impairment, Sensory loss

## Abstract

The purpose of this study is to evaluate the association between perinatal asphyxia, neonatal encephalopathy, and childhood hearing impairment. This is a population-based study including all Norwegian infants born ≥ 36 weeks gestation between 1999 and 2014 and alive at 2 years (*n* = 866,232). Data was linked from five national health registries with follow-up through 2019. Perinatal asphyxia was defined as need for neonatal intensive care unit (NICU) admission and an Apgar 5-min score of 4–6 (moderate) or 0–3 (severe). We coined infants with seizures and an Apgar 5-min score < 7 as neonatal encephalopathy with seizures. Infants who received therapeutic hypothermia were considered to have moderate-severe hypoxic-ischemic encephalopathy (HIE). The reference group for comparisons were non-admitted infants with Apgar 5-min score ≥ 7. We used logistic regression models and present data as adjusted odds ratios (aORs) with 95% confidence intervals (CI). The aOR for hearing impairment was increased in all infants admitted to NICU: moderate asphyxia aOR 2.2 (95% CI 1.7–2.9), severe asphyxia aOR 5.2 (95% CI 3.6–7.5), neonatal encephalopathy with seizures aOR 7.0 (95% CI 2.6–19.0), and moderate-severe HIE aOR 10.7 (95% CI 5.3–22.0). However, non-admitted infants with Apgar 5-min scores < 7 did not have increased OR of hearing impairment. The aOR for hearing impairment for individual Apgar 5-min scores in NICU infants increased with decreasing Apgar scores and was 13.6 (95% CI 5.9–31.3) when the score was 0.

*          Conclusions*: An Apgar 5-min score < 7 in combination with NICU admission is an independent risk factor for hearing impairment. Children with moderate-severe HIE had the highest risk for hearing impairment.

**Table:** 

**What is Known:** *• Perinatal asphyxia and neonatal encephalopathy are associated with an increased risk of hearing impairment.* *• The strength of the association, and how other co-morbidities affect the risk of hearing impairment, is poorly defined.*	
**What is New:** *• Among neonates admitted to a neonatal intensive care unit (NICU), decreased Apgar 5-min scores, and increased severity of neonatal encephalopathy, were associated with a gradual rise in risk of hearing impairment.* *• Neonates with an Apgar 5-min score 7, but without NICU admission, did not have an increased risk of hearing impairment.*

**Supplementary Information:**

The online version contains supplementary material available at 10.1007/s00431-023-05321-5.

## Introduction

Childhood hearing impairment is a matter of public health interest because of both high prevalence and the potential negative impact on language and general development if not early and correctly diagnosed and treated [1, 2]. Perinatal asphyxia and neonatal encephalopathy may cause long-term disability among survivors [3–6] and are risk factors for sensorineural hearing impairment [7–9]. The reported risk of later hearing impairment in neonates exposed to asphyxia and neonatal encephalopathy varies between studies [5, 10, 11] and depends on prenatal susceptibility, co-morbidities, and exposures to other potential harmful or protective therapies [8, 9]. Increased understanding of the etiology of childhood hearing impairment is important for preventive measures, prognostic evaluation, and to identify high-risk populations for targeted surveillance [7].

A clinical diagnosis of perinatal asphyxia is optimally based upon documented impaired gas exchange of the fetus[12]. The Apgar score alone is not a diagnostic tool for asphyxia, but the majority of scores less than seven after 5 min may still be attributed to asphyxia, and low Apgar scores are correlated to cord blood gas acidosis [12–14]. In epidemiological studies, a low Apgar score has a high predictive value for later development of cerebral palsy and epilepsy [4, 15, 16]. Population-based studies have also shown an increasing risk of hearing impairment with decreasing Apgar scores, but often without adequate correction for possible confounders [9, 17]. The introduction of therapeutic hypothermia for neonates with moderate-severe neonatal encephalopathy of presumed hypoxic-ischemic origin has reduced mortality and long-term disability in survivors [6, 18]. However, the prevalence of hearing impairment in this population is still high [6, 10, 18, 19], and it is unclear if therapeutic hypothermia can modify this outcome [6, 18]. Contemporary information regarding rates of hearing impairment, risk factors, and associated morbidities among neonates with perinatal asphyxia and neonatal encephalopathy are needed.

In this population-based registry study, we aimed to identify the association between perinatal asphyxia, neonatal encephalopathy, other neonatal morbidities, and sensorineural hearing impairment in an unselected population of all Norwegian infants born ≥ 36 weeks gestation in the 16-year period 1999–2014, and with follow-up data on diagnosed hearing impairment for all participants through 2019.

## Methods

### Data sources, setting, and study approval

We collected individual-level data on study participants from five Norwegian national health and social registries (Supplementary Table 1). Data were linked using the unique Norwegian 11-digit personal identification numbers of the infant and the mother. The Medical Birth Registry of Norway (MBRN) has almost complete prospectively collected data on pregnancy, delivery, and maternal and neonatal health until 12 months of age [20, 21]. The Norwegian Patient Registry (NPR) contains diagnoses (International Classification of Diseases, 10th Revision-ICD-10) and surgical and medical procedural codes reported from health care providers in both public and private specialist-health care sectors since its inception in 2008 [22, 23]. Reimbursement for inpatient and outpatient visits is based on automatic reports to the NPR, providing a high completeness of data. We included in this study NPR data from 2008 through 2019. The Norwegian National Insurance Scheme (NIS) is the public social security system in Norway with data on diagnoses for illness-related expenses, including people suffering from hearing impairment [24]. Financial compensation is without regard to wealth or income, and virtually everyone with a diagnosis entitled to financial benefit is registered in the NIS. We included data from NIS through 2019. The Norwegian Cause of Death registry contains time and cause of death according to ICD-10 diagnoses [25]. Statistics Norway contains data on parental immigration status and the educational level of the mother.

Universal newborn hearing screening was established in Norway in 2008. The study and linkage between the five registries were approved by the Regional Ethical Committee for medical and health research ethics (REK nr. 2018/1789).

### Study population—inclusion and exclusion criteria

**Fig. 1:**
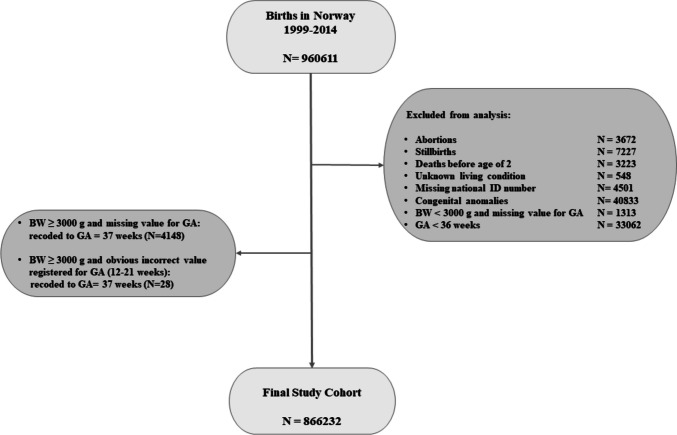
Study cohort flow diagram. BW birth weight, GA gestational age, ID identification

Our target population, identified from the MBRN, were infants born ≥ 36 completed weeks gestation in Norway in the 16-year period 1999–2014 (Fig. 1). We excluded children who died during the first 2 years of life due to insufficient follow-up time for hearing impairment diagnosis. We also excluded children with congenital malformations, considered a major confounding factor for the association between asphyxia and hearing impairment [2]. Preterm infants less than 36 weeks gestation were excluded to remove the influence of prematurity complications associated with increased rates of hearing impairment [26]. Among children with missing or obviously incorrect data for gestational age (GA) at birth, we included those with birthweight ≥ 3000 g and alive at 2 years of age and pragmatically assigned them a GA of 37 weeks.

### Exposures

We defined six different exposure groups and a healthy reference group consisting of non-admitted infants with an Apgar 5-min score ≥ 7 (Table 1). In Norway, cord blood gas analyses are not the routinely taken except in high-risk deliveries, and data were not available from the MBRN.

**Table 1:** Infants born ≥ 36 weeks gestation in Norway 1999–2014 and alive at 2 years of age. Distribution of maternal and infant characteristics in groups according to severity of perinatal asphyxia and neonatal encephalopathy and other morbidities

**Total cohort** ***N* = 866,232**	**Reference group** ***N* = 759,322**	**Group 1** ***N* = 53,572**	**Group 2** ***N* = 2175**	**Group 3** ***N* = 4591**	**Group 4** ***N* = 972**	Group 5*** N = 115	Group 6*** N = 155
Criteria for group definition	Apgar 5-min 7–10 No NICU admission	Apgar 5-min 7–10 NICU-admission	Apgar 5-min < 7 No NICU admission	Apgar 5-min 4–6 NICU-admission	Apgar 5-min 0–3 NICU-admission	Apgar 5-min < 7 NICU-admission Seizures, no TH	Received therapeutic hypothermia (TH)
Clinical description	**Healthy**	**Neonatal illness—not asphyxia**	**Low Apgar score, rapid recovery**	**Moderate asphyxia**	**Severe asphyxia**	**Neonatal encephalopathy with seizures**	**Moderate-severe HIE**
**Maternal characteristics**							
Daily smoking early in pregnancy*	83,906 (13.1)	7426 (16.5)	266 (14.6)	520 (13.8)	104 (13.1)	19 (18.6)	10 (7.2)
Low education (high school or less)	340,518 (44.8)	26,575 (49.6)	1022 (47.0)	2159 (47.0)	489 (50.3)	59 (51.3)	67 (43.2)
Body mass index ≥ 30 **	22,099 (12.0)	2323 (17.4)	78 (14.2)	210 (19.6)	42 (17.4)	9 (20.0)	15 (20.0)
Parental consanguinity	7646 (1.0)	597 (1.1)	36 (1.7)	44 (1.0)	11 (1.1)	1 (0.9)	0
Immigrant	94,186 (12.4)	6633 (12.4)	303 (13.9)	658 (14.3)	141 (14.5)	21 (18.3)	26 (16.8)
Emergency cesarean delivery	57,481 (7.6)	11,089 (20.7)	310 (14.3)	1410 (30.7)	356 (36.6)	36 (31.3)	58 (37.4)
**Infant characteristics**							
Mean (SD) birth weight (g)	3584 (494)	3472(691)	3611 (567)	3548 (618)	3580 (634)	3568 (593)	3643 (617)
Mean (SD) head circumference (cm)	35 (1.6)	35 (1.9)	35 (1.6)	35.4 (1.8)	35.5 (1.6)	35.4 (1.6)	35.4 (1.5)
Median (IQR) gestational age (weeks)	40 (39–41)	39 (38–40)	40 (39–41)	40 (39–41)	40 (39–41)	40 (39–41)	40 (39–41)
Male	383,553 (50.5)	30,784 (57.5)	1243 (57.1)	2598 (56.6)	536 (55.1)	63 (54.8)	84 (54.2)
Small for gestational age	62,871 (8.3)	8975 (16.8)	257 (11.8)	709 (15.4)	164 (16.9)	22 (19.1%)	21 (13.5)
Jaundice therapy	29,244 (3.9)	10,933 (20.4)	117 (5.4)	404 (8.8)	63 (6.5)	4 (3.5)	3 (1.9)
Antibiotic therapy	NA	13,498 (25.2)	NA	1780 (38.8)	561 (57.7)	93 (80.9)	150 (96.8)
Non-invasive respiratory support	NA	3683 (6.9)	NA	980 (21.3)	251 (25.8)	38 (33)	64 (41.3)
Mechanical ventilation	NA	464 (0.9)	NA	386 (8.4)	319 (32.8)	45 (39.1)	127 (80.9)

Data are number and proportions (%), unless otherwise stated

*HIE* hypoxic-ischemic encephalopathy, *min* minute, *NA* not applicable, *NICU* neonatal intensive care unit, *TH* therapeutic hypothermia

* Missing data for 15.7% of population; **Missing data for 77% of population; *** Data available for birth cohorts 2008–2014 only (*N* = 391,817)

The main exposure was perinatal asphyxia, defined as the need for neonatal intensive care unit (NICU) admission in combination with an Apgar 5-min score below 7 [27], and further subclassified into a moderate group and a severe group with 5-min scores of 4–6 and 0–3, respectively. We considered the need for NICU admission in these term/near-term infants as a sign of neonatal compromise with symptoms requiring observation and/or therapy. The group of infants with Apgar 5-min scores < 7 and not admitted to a NICU were considered to have had a “rapid recovery” and not classified as perinatal asphyxia. Infants admitted to a NICU with Apgar 5-min scores ≥ 7 were defined in a separate exposure group classified as “other neonatal morbidity.” For these four groups, data was available for the entire 16-year cohort.

Neonatal encephalopathy was a secondary exposure and was subclassified in two separate groups. The first group was coined neonatal encephalopathy with seizures and defined as newborn infants admitted to a NICU with an Apgar 5-min score < 7 and diagnosed with neonatal seizures, but not receiving therapeutic hypothermia. The second group was coined moderate-severe HIE and included infants that received therapeutic hypothermia after inclusion criteria specified in the original TOBY study protocol [28]. Data on neonatal encephalopathy was based on diagnoses and therapies registered in the NPR and therefore only available for the 2008–2014 birth cohort.

In addition to analyses of hearing impairment in these six specific exposure groups, we analyzed the association between individual Apgar scores in NICU-admitted children and their subsequent risk of hearing impairment.

### Confounders, mediators, and covariates

GA was determined by second-trimester fetal ultrasound and, if missing, by the last menstrual period. Other maternal variables included in the analysis were body mass index (BMI), daily smoking early in pregnancy, immigrant status, parental consanguinity, educational level, and mode of delivery. Neonatal variables, obtained from the MBRN or NPR, were infant sex, birthweight, head circumference, small for gestational age (SGA) defined as birthweight < 10 percentile for GA, antibiotic therapy, neonatal sepsis (ICD-10 code P36 newborn sepsis), jaundice therapy, non-invasive respiratory support, and mechanical ventilation.

### Outcomes and definitions

The main outcome of this study, sensorineural hearing impairment, was defined by selected ICD-10 codes for hearing impairment retrieved from two registries (NPR and NIS). From NPR, we included patients registered with the ICD-10 codes H90.3–5 for sensorineural hearing impairment. To reduce false positive cases due to possible coding errors, one of the ICD-10 codes H90.3–5, or a combination of these codes, had to be registered a minimum of two times in the NPR before we considered that the patient had a definite diagnosis of sensorineural hearing impairment. Patients with a diagnosis of conductive, mixed, or unspecified hearing loss registered in the NPR were not included as these are mainly related to middle ear disease, genetic syndromes, or craniofacial deformities and not associated with hypoxic damage to the cochlea or central auditory system, which is the focus of this study. In order to capture cases from two independent registries, we also identified patients with hearing impairment diagnosis from the NIS. As expected, a much lower number of patients were identified in the NIS. Moreover, an explorative analysis of ICD-10 diagnoses in the NIS showed that most codes for hearing impairment were coded as “unspecific,” probably reflecting a focus on the degree of disability and not diagnostic code accuracy. From NIS, we therefore added all patients registered with “H90 Conductive and sensorineural hearing loss” and “H91 Other and unspecified hearing loss.” A complete list of the ICD-10 diagnoses for hearing impairment and the number of cases included from the NPR and the NIS is displayed in Supplementary Table 2.

### Statistical methods

**Fig. 2:**
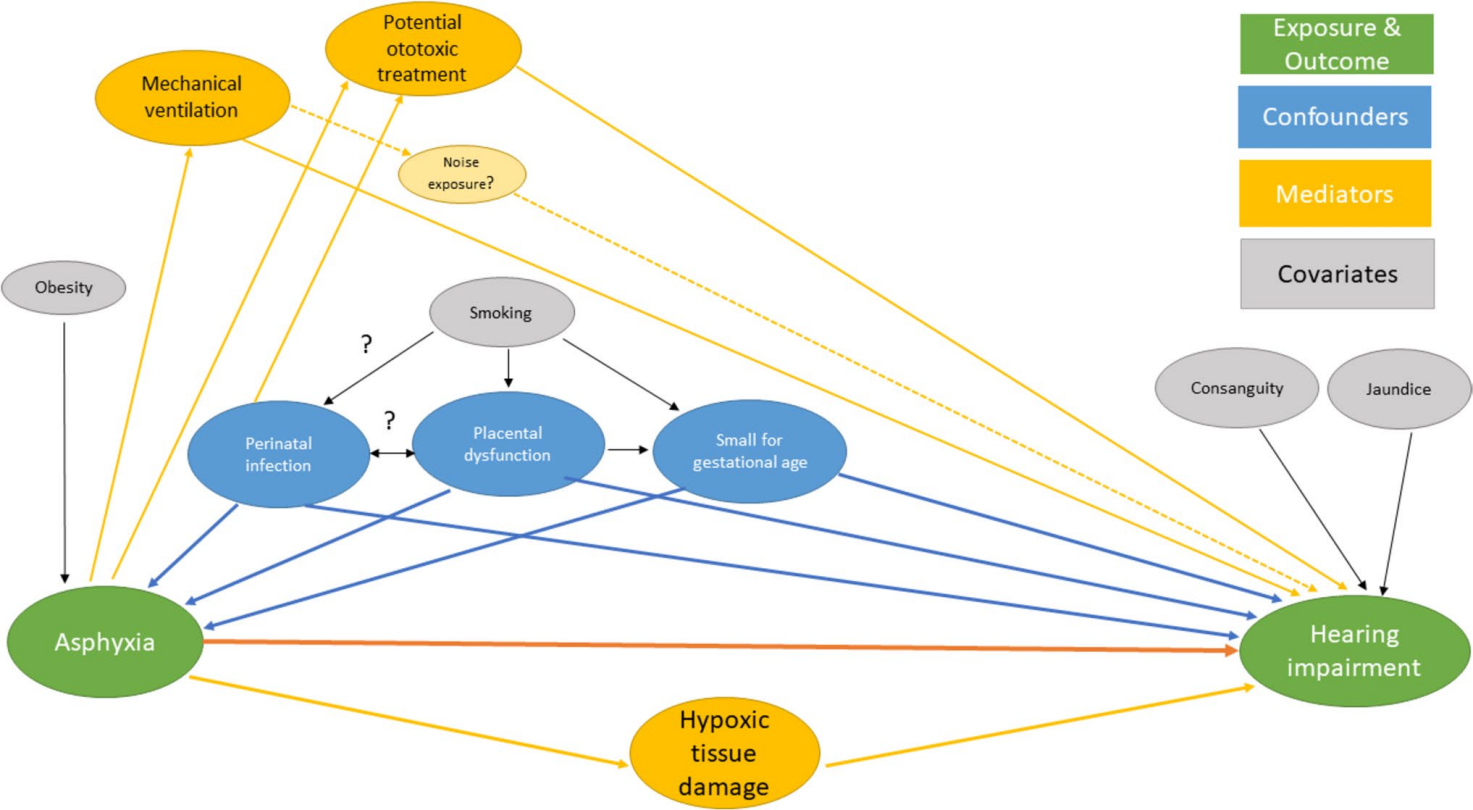
Directed acyclic graph on associations between perinatal asphyxia and hearing impairment

We used the SPSS software (28.0.1.0) for all statistical analyses. Results are presented as proportions, means with standard deviations (SD), or medians with interquartile range (IQR), as appropriate. To evaluate the association between sensorineural hearing impairment and the exposure groups of interest, we used logistic regression analysis with reference to the group of children that had an Apgar 5-min score between 7 and 10 points and no record of a NICU admission. For the analysis of individual Apgar 5-min score values, we used non-admitted children with an Apgar 5-min score equal to 10 as a reference. Based on previous reports [8, 9], and by drawing directed acyclic graphs (DAGs) (Fig. 2), we identified possible confounders as fetal growth restriction and perinatal infection. We found it valid to use the variable SGA as a marker for growth restriction and adjusted for this in our main analysis. Data for antibiotic use (in birth cohorts 1999–2014) and both culture-proven and culture-negative neonatal sepsis (in birth cohorts 2008–2014) were available (Table 1). However, these variables would overestimate the true burden of severe perinatal infection and potentially lead to overcorrection. Thus, we only used them, in addition to SGA, for adjustment in an explorative analysis. The main analysis was repeated with interaction term to investigate possible differences between boys and girls. Crude and adjusted odds ratios (ORs) are presented with a 95% confidence interval (CI).

## Results

From January 1, 1999, through December 31, 2014, 960,611 births with GA ≥ 36 weeks were registered in Norway. After exclusions, the final study cohort constituted 866,232 children (Fig. 1). Apgar 5-min scores were available for 864,944 (99.9%) of the study participants. Maternal and infant characteristics are displayed in Table 1. In the final study cohort, 7845 (0.9%) of all newborn infants had an Apgar 5-min score < 7, and among these, 5563 (70.9%) infants were admitted to a NICU. Among admitted infants with Apgar 5-min score 0–3 or infants with neonatal encephalopathy, antibiotic therapy and mechanical ventilation were markedly more common than for all other groups. Boys were overrepresented in all groups admitted to a NICU and among non-admitted newborn infants with an Apgar 5-min score < 7.

The prevalence of sensorineural hearing impairment, diagnosed after a minimum 5-year follow-up, in healthy newborn infants with an Apgar 5-min score of 7–10 and not admitted to a NICU (*n* = 759,322) was 0.6%, equal in boys and girls. The prevalence of sensorineural hearing impairment was increased in all infants admitted to a NICU (Table 2), but not significantly different between boys and girls with perinatal asphyxia (Supplementary Table 3). The severity of perinatal asphyxia was associated with a higher prevalence of sensorineural hearing impairment, and it was highest among infants with moderate-severe HIE. However, infants with an Apgar 5-min score < 7, but not admitted to a NICU, did not have an increased prevalence of sensorineural hearing impairment. Results remained similar in exploratory analyses adjusting also for antibiotic therapy/sepsis (Supplementary Table 4). Table 3 displays crude and adjusted ORs for sensorineural hearing impairment for individual Apgar 5-min score values among infants admitted to a NICU, with reference to non-admitted infants with an Apgar 5-min score of 10. The ORs increased markedly with decreasing Apgar 5-min values, and the adjusted OR (aOR) was 13.6 (95% CI 5.9–31.3) in infants with an Apgar 5-min score of 0. We also analyzed the Apgar 5-min scores as a continuous variable, and each unit decrease in the Apgar 5-min score was associated with an aOR of 1.15 (95% CI 1.13–1.17) of sensorineural hearing impairment.

**Table 2:**
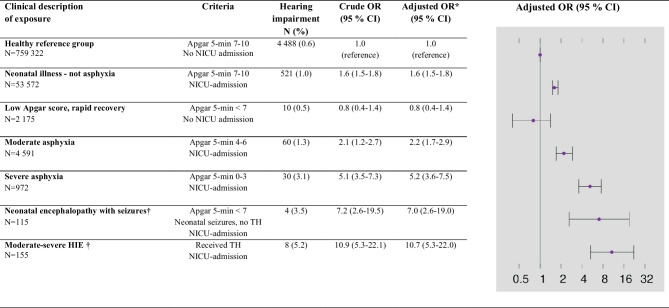
Prevalence, crude, and adjusted odds ratio (OR) for sensorineural hearing impairment, in relation to perinatal asphyxia and neonatal morbidity, among 866,232 infants born ≥ 36 weeks gestation in Norway 1999–2014 and alive at 2 years of age

*HIE* hypoxic-ischemic encephalopathy, *min* minute, *NICU *neonatal intensive care unit, *TH* therapeutic hypothermia

^*^ Adjusted for being small for gestational age

^a^ Data available only for birth cohorts 2008–2014 (*N* = 391,817)

**Table 3:**
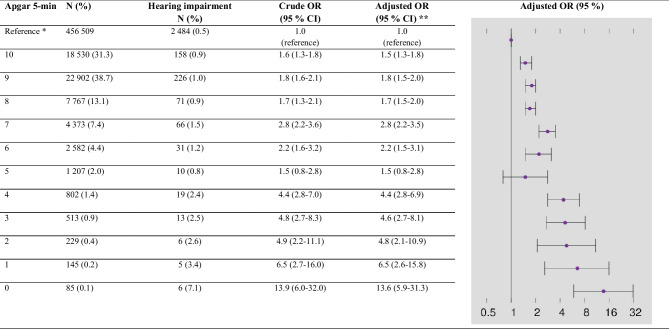
Apgar 5-min scores, crude, and adjusted odds ratio (OR) for sensorineural hearing impairment among 59,135 infants born ≥ 36 gestation and admitted to NICU in Norway 1999–2014 and alive at 2 years of age

*NICU* neonatal intensive care unit, *min* minute

^*^ The reference was children with Apgar 5-min = 10 and not admitted to a NICU; ** Adjusted for a diagnosis of being small for gestational age

In Supplementary Table 5, we present a separate analysis for confounders, covariates, and mediators. After adjustment for severe perinatal asphyxia, the variables that remained associated with an increased aOR (95% CI) for sensorineural hearing impairment were neonatal mechanical ventilation 2.9 (1.4–5.9), being SGA 1.3 (1.2–1.4), neonatal jaundice therapy 1.2 (1.1–1.4), parental consanguinity 1.9 (1.5–2.3), daily smoking early in pregnancy 1.3 (1.2–1.5), and maternal low education 1.2 (1.2–1.3).

## Discussion

In this population-based study with an unselected cohort of more than 866,000 infants born ≥ 36 weeks gestation, we found that perinatal asphyxia, defined as an Apgar 5-min score < 7 and need for NICU admission, was an independent risk factor for sensorineural hearing impairment. In line with others [17], we observed an inverse relation between the Apgar 5-min scores and the later risk of sensorineural hearing impairment. The highest risk of sensorineural hearing impairment was observed among babies who had moderate-severe HIE. In contrast, we found that Apgar 5-min scores < 7 in infants not admitted to a NICU had no increased risk for sensorineural hearing impairment.

Studies investigating the associations between low Apgar scores and hearing impairment [29] report conflicting results [9, 17, 30–32]. In a study of 11,000 infants, an Apgar 5-min score < 7 was not associated with hearing screening failure at NICU discharge, but the study lacked data on neonatal encephalopathy [30]. In contrast, in a smaller study including only “at risk” infants, an Apgar 5-min score < 7 was a significant risk factor for hearing screening failure, but without adjustments for other risk factors, including prematurity [31]. The inconsistent results are probably due to differences in populations including infants with co-morbidities like prematurity and birth malformations that may affect the predictive value of the Apgar score [15]. Moreover, a newborn hearing screening result does not represent a permanent hearing loss, and delayed onset hearing loss is reported to be more frequent in children exposed to asphyxia or intensive care therapy [7]. A Norwegian study on infants born between 1978 and 1998 found a similar increase in ORs for permanent hearing impairment in relation to low Apgar score as in our study [17]. Another population-based study with a 3-year follow-up of around 115,000 babies found that 8.9% of children with Apgar 5-min score < 7 was later diagnosed with permanent hearing impairment, corresponding to an OR of 20. Notably, half of them were of delayed-onset and diagnosed after the newborn hearing screen [9]. The much higher prevalence and risk for hearing impairment found in this latter study could be explained by also including preterm infants and not adjusting for confounding morbidities.

A salient finding in our study was the lack of association between Apgar 5-min scores < 7 and sensorineural hearing impairment in children with no history of NICU admission. This may have different explanations. Infants not admitted to a NICU did probably not have other co-morbidities, and several studies indicate that the synergistic effect of several harmful exposures is more important than a low Apgar score alone [9, 17, 30–32]. Also, the inner ear and cochlear structures may be more resilient to hypoxia compared with other parts of the brain. Studies on associations between Apgar 5-min scores and cerebral palsy [4, 16] report much higher ORs for cerebral palsy at the same Apgar 5-min score levels than the ORs for sensorineural hearing impairment in our study, and this may support our theory.

We found a high prevalence of sensorineural hearing impairment (5.2%) in infants who had received therapeutic hypothermia, in line with others reporting prevalences between 3.8 and 10%.[6, 10, 18, 19]. Both a Cochrane review on therapeutic hypothermia [18] and follow-up studies in school age [6, 33] report lower absolute numbers of hearing impairment in cooled infants compared with normothermia, but not reaching significance, which could be due to small samples. Our study was not designed to assess a potential protective effect of cooling for hearing impairment. However, the effect of hypothermia on the inner ear has been thoroughly evaluated in animal studies in which hypothermia had significant and potentially clinically meaningful otoprotection [34].

The precise pathophysiological mechanisms behind hearing impairment caused by perinatal asphyxia and hypoxia remain unclear. Both clinical studies with objective hearing tests [35] and postmortem pathological studies [36] indicate that hypoxia may damage both hair cells of the cochlea and affect retrocochlear auditory function [37]. There is also evidence that synergistic effects between asphyxia and other insults, like jaundice, may play a role in the pathogenesis [38]. A study on cochlear hair cell function in infants exposed to mild and moderate asphyxia found that they had significantly reduced otoacoustic activity in cochlear hair cells compared to controls, even though they had passed the newborn hearing screening [39]. Thus, an early hypoxic event can impose an increased risk for delayed-onset hearing impairment.

Our study adds information for the design of targeted hearing surveillance and intervention programs, based on risk factors for hearing impairment [9]. The Joint Committee of Infant Hearing has regularly updated their risk factors, and therapeutic hypothermia was included in the latest position statement from 2019 [7]. Based on data from our study, an Apgar 5-min score < 4 and admission to a NICU or clinical signs of neonatal encephalopathy are strong risk factors for later hearing impairment. In contrast, a more moderate perinatal asphyxia and/or low Apgar scores alone without the need for NICU admission is not associated with high risk.

The strength of this study is that it includes a large, population-based national cohort. We used data from several validated national registries to identify the vast majority of individuals with hearing impairment and to include relevant confounders. We used DAGs to select confounders for the logistic regression analysis, but cannot exclude residual confounding. If the Apgar 5-min score is < 7, it is recommended that an infant should be observed closely [12]. We therefore added the need for NICU admission in our definition of perinatal asphyxia. Lack of cord blood gas data is a limitation with our study. Still, in other studies, authors have reported consistent associations between low Apgar scores and other adverse neurodevelopmental outcomes [4, 16]. Moreover, cord blood gas acidosis is not a good predictor of perinatal asphyxia [14]. Recent studies from the UK and Sweden reported that Apgar 5-min scores below 7 had a higher predictive value for HIE development than a low pH in the cord blood [40, 41]. Our study could not assess the degree of hearing impairment as the ICD-10 codes for hearing impairment do not include criteria for hearing level in decibel (dB), frequency range, or methods for hearing measurement. The overall prevalence of sensorineural hearing impairment in our study population was somewhat higher than reported from other Scandinavian studies [42, 43]. This may be due to different criteria used for diagnosing sensorineural hearing impairment, and that the use of ICD-10 codes may overestimate cases of hearing impairment. However, this will not affect our calculated ORs for different risk groups. Finally, procedural codes for hearing aids and cochlear implants were unfortunately not available for this study. They would have provided a better definition of severe hearing impairment and its impact on patient lives.

## Conclusion

An Apgar 5-min score < 7 in combination with a NICU admission was an independent risk factor for hearing impairment in this unselected cohort of 866,000 children. The risk of hearing impairment increased by lower Apgar scores and was highest in children with moderate-severe HIE. Our study contributes with data on risk factors for hearing impairment that can be used for targeted early hearing surveillance, intervention, and follow-up programs.

### Supplementary Information

Below is the link to the electronic supplementary material.

Supplementary file1 (DOCX 84 KB)

## Data Availability

The study has utilized Norwegian governmental registries and legal restrictions do not permit the authors to share these data.

## Abbreviations

BMI: Body mass index
BW: Birth weight
GA: Gestational age
HIE: Hypoxic-ischemic encephalopathy
MBRN: Medical Birth Registry of Norway
NICU: Neonatal intensive care unit
NPR: Norwegian Patient Registry
SGA: Small for gestational age
