# Supplementary material for: Hearing impairment after asphyxia and neonatal encephalopathy: a Norwegian population-based study
Source: Eur J Pediatr. 2023 Nov 22;183(3):1163–72. doi: 10.1007/s00431-023-05321-5 (PMC10950958; doi:10.1007/s00431-023-05321-5)
Supplement: Supplementary file 1 — Supplementary file1 (DOCX 84 KB) [file 431_2023_5321_MOESM1_ESM.docx]

**Supplementary Table 1**. Description of five Norwegian national health and social registries providing data for this study.

| **Registries** | **Description** | **Main variables used in the study** | **Data available from years** |
| --- | --- | --- | --- |
| Medical Birth Registry of Norway | Provides data on pregnancy, delivery, and perinatal period. | Maternal health variables, mode of delivery, gestational age, birth weight, Apgar scores, congenital malformations, admission data and basic treatment variables in the neonatal period, see Table 1. | 1999- 2019 |
| The Norwegian Patient Registry | Provides data on diagnostic and surgical and medical procedural codes, | Detailed diagnoses for hearing impairment and other relevant diagnoses and therapies used in the NICU, see Table 1. | 2008-2019 |
| The Norwegian National Insurance Scheme | Provides data on diagnoses causing illness-related expenses | Only hearing impairment | 1999-2019 |
| Norwegian Cause of Death registry | Provides time and cause of death according to ICD-10 diagnoses | Data was used to filter out participants that died before the age of 2 years. | 1999-2019 |
| Statistics Norway | Provides data on a wealth of aspects from national statistics in Norway | Data on parental immigration status and educational level of the mother | 1999-2019 |

**Supplementary Table 2**. Diagnostic codes for hearing impairment in ICD-10 and included cases from Norwegian patient registry (NPR) and the Norwegian national insurance scheme (NIS).

| **ICD-10**  **Diagnostic codes** |  | **Cases included from NPR*** | **Cases included from NIS**** | **Cases registered in both NPR and NIS** | **Total number of cases included.** |
| --- | --- | --- | --- | --- | --- |
| H 90.0 | Conductive hearing loss, bilateral | No | No |  |  |
| H 90.1 | Conductive hearing loss, unilateral | No | No |  |  |
| H 90.2 | Conductive hearing loss, unspecified | No | No |  |  |
| H 90.3 | Sensorineural hearing loss, bilateral | Yes | Yes |  |  |
| H 90.4 | Sensorineural hearing loss, unilateral | Yes | Yes |  |  |
| H 90.5 | Sensorineural hearing loss, unspecified | Yes | Yes |  |  |
| H 90.6 | Mixed conductive and sensorineural hearing loss, bilateral | No | Yes |  |  |
| H 90.7 | Mixed conductive and sensorineural hearing loss, unilateral | No | Yes |  |  |
| H 90.8 | Mixed conductive and sensorineural hearing loss, unspecified | No | Yes |  |  |
| H 91.0 | Ototoxic hearing loss | No | No |  |  |
| H 91.1 | Presbycusis | No | No |  |  |
| H 91.2 | Sudden idiopathic hearing loss | No | No |  |  |
| H 91.3 | Deaf mutism, not elsewhere classified | No | Yes |  |  |
| H 91.8 | Other specified hearing loss | No | Yes |  |  |
| H 91.9 | Hearing loss, unspecified | No | Yes |  |  |
| **TOTAL** |  | 5 339 | 433 | 363 | 5 409 |

* if registered two times or more
** if registered once or more

**Supplementary Table 3.** Prevalence, crude and adjusted odds ratio (OR) for sensorineural hearing impairment, in relation to perinatal asphyxia and neonatal morbidity, among boys and girls in a total cohort of 866 232 infants born ≥ 36 weeks gestation between 1999 and 2014 and alive at 2 years of age.

|  | | | **BOYS** | **GIRLS** | **BOYS (1)** | **GIRLS (0)** | **Interaction term** |
| --- | --- | --- | --- | --- | --- | --- | --- |
| **Gender distribution within group** | **Criteria for group definition** | **Clinical description** | **Hearing impairment**  **N %** | **Hearing impairment**  **N (%)** | **Adjusted OR** *  **(95% CI)** | **Adjusted OR** *  **(95% CI)** | **Adjusted OR** *  **(95% CI)** |
| Reference group  N=759 322  Boys 50.5% (N= 383 553) | Apgar 5-min 7-10  No NICU-admission | **Healthy** | 2301 (0.6) | 2187 (0.6) | Not applicable | Not applicable | Not applicable |
| Group 1  N=53 572  Boys 57.5% (N= 30 784) | Apgar 5-min 7-10  NICU-admission | **Neonatal illness - not asphyxia** | 298 (1) | 223 (1) | 1.6 (1.4-1.8) | 1.6 (1.4-1.9) | **1.6 (1.4-1.8)** |
| Group 2  N=2 175  Boys 57.1 % (N=1 243) | Apgar 5-min < 7  No NICU-admission | **Low Apgar score, rapid recovery** | 8 (0.6) | 2 (0.2) | 1.1 (0.5-2.1) | 0.4 (0.1-1.5) | 3.0 (0.6-14.2) |
| Group 3  N=4 591  Boys 56.6 % (N=2 598) | Apgar 5-min: 4-6  NICU-admission | **Moderate asphyxia** | 38 (1.5) | 22 (1.1) | 2.4 (1.7-3.3) | 1.9 (1.2-2.9) | 1.3 (0.8-2.2) |
| Group 4  N=972  Boys 55.1 % (N=536) | Apgar 5-min: 0-3  NICU-admission | **Severe asphyxia** | 19 (3.5) | 11 (2.5) | 5.9 (3.7-9.4) | 4.3 (2.4-7.9) | 1.4 (0.7-3.0) |
| Group 5**†**  N=115  Boys 54.8 % (N=63) | Apgar 5-min < 7  NICU-admission Seizures, no TH | **Neonatal encephalopathy with seizures†** | 3 (4.8) | 1 (1.9) | 9.1 (2.8-29.1) | 4.1 (0.6-29.9) | 2.6 (0.3- 25.3) |
| Group 6**†**  N=155  Boys 54.2 % (N=84) | Moderate-severe HIE  NICU-admission TH | **Moderate-severe HIE and TH†** | 6 (7.1) | 2 (2.8) | 14.2 (6.2-32.6) | 6.2 (1.5-25.1) | 2.6 (0.5-13.5) |

**†** Data available only for birth cohorts 2008-2014 (N = 391 817)

*Adjusted for a diagnosis of being small for gestational age

HIE; hypoxic-ischemic encephalopathy, NICU; neonatal intensive care unit, TH; therapeutic hypothermia

**Supplementary Table 4.** Prevalence, crude, and adjusted odds ratio (OR) for sensorineural hearing impairment, in relation to perinatal asphyxia and neonatal morbidity, among 866 232 infants born ≥ 36 weeks gestation in Norway 1999-2014 and alive at two years of age.

| **Clinical description**  **of exposure** | **Criteria** | **Hearing impairment**  **N (%)** | **Crude OR**  **(95 % CI)** | **Adjusted OR**  **(95 % CI)** | **Adjusted OR (95 % CI)**  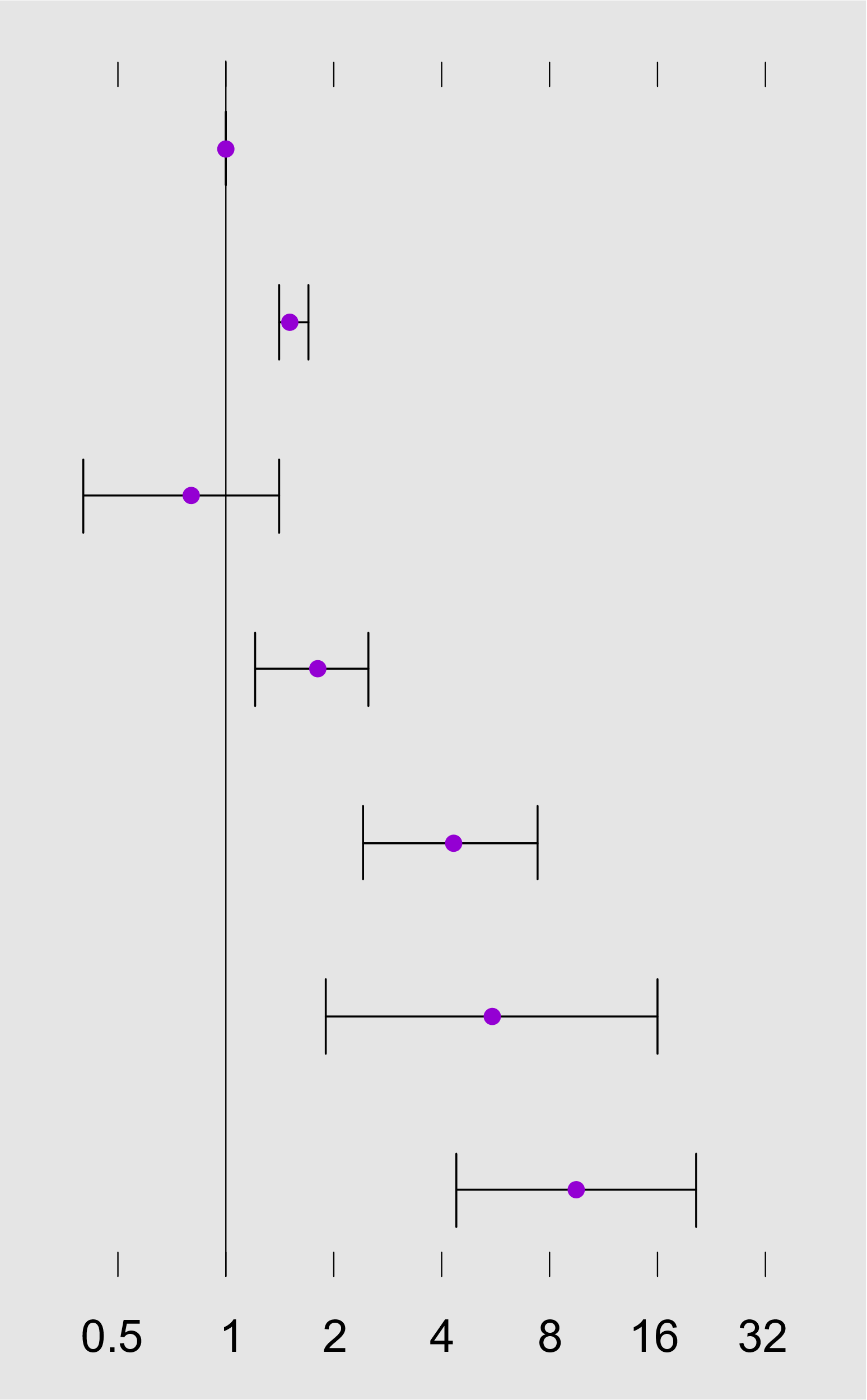 |
| --- | --- | --- | --- | --- | --- |
| **Healthy reference group**  N=759 322 | Apgar 5-min 7-10  No NICU admission | 4 488 (0.6) | 1.0  (reference) | 1.0  (reference) |  |
| **Neonatal illness - not asphyxia**  N=53 572 | Apgar 5-min 7-10  NICU-admission | 521 (1.0) | 1.6 (1.5-1.8) | 1.5 (1.4-1.7)  ** |  |
| **Low Apgar score, rapid recovery**  N=2 175 | Apgar 5-min < 7  No NICU admission | 10 (0.5) | 0.8 (0.4-1.4) | 0.8 (0.4-1.4)  ** |  |
| **Moderate asphyxia**  N=4 591 | Apgar 5-min 4-6  NICU-admission | 60 (1.3) | 2.1 (1.2-2.7) | 1.8 (1.2-2.5)  ** |  |
| **Severe asphyxia**  N=972 | Apgar 5-min 0-3  NICU-admission | 30 (3.1) | 5.1 (3.5-7.3) | 4.3 (2.4-7.4)  ** |  |
| **Neonatal encephalopathy †**  N=115 | Apgar 5-min < 7  Neonatal seizures, no TH  NICU-admission | 4 (3.5) | 7.2 (2.6-19.5) | 5.5 (1.9-16.0) *** |  |
| **Moderate-severe HIE †**  N=155 | Received TH  NICU-admission | 8 (5.2) | 10.9 (5.3-22.1) | 9.5 (4.4-20.4) *** |  |

**†** Data available only for birth cohorts 2008-2014 (N = 391 817)
** Adjusted for neonatal systemic antibiotic therapy and a diagnosis of being small for gestational age
*** Adjusted for being diagnosed with neonatal sepsis and a diagnosis of being small for gestational age
HIE; hypoxic-ischemic encephalopathy, min; minute, NICU; neonatal intensive care unit, TH; therapeutic hypothermia.

**Supplementary Table 5**. Prevalence, crude, and adjusted odds ratio (OR) for sensorineural hearing impairment in relation to defined confounders, mediators, and covariates among 866 232 infants born ≥ 36 weeks gestation in Norway 1999-2014.

| **EXPOSURES** | **N (%)** | **Hearing impairment N (%)** | **Crude OR (95 % CI)** | **Adjusted OR (95 % CI) *** |
| --- | --- | --- | --- | --- |
| **Confounders** | | | | |
| Antibiotic therapy | 16 196 (1.9) | 200 (1.2) | 2.0 (1.8-2.3) | 1.4 (0.7-2.7) |
| Neonatal sepsis **†** | 2 976 (0.8) | 38 (1.3) | 2.5 (1.8-3.4) | 1.7 (0.6-4.8) |
| Small for gestational age | 76 858 (8.9) | 645 (0.8) | 1.4 (1.3-1.5) | **1.3 (1.2-1.4)** |
| **Mediators** | | | | |
| Mechanical ventilation | 1 220 (0.1) | 53 (4.3) | 7.3 (5.5-9.6) | **2.9 (1.4-5.9)** |
| Non-invasive respiratory support | 5 045 (0.6) | 78 (1.5) | 2.5 (2.0-3.2) | 1.7 (0.8-3.5) |
| **Covariates** | | | | |
| Jaundice therapy | 42 555 (4.9) | 338 (0.8) | 1.3 (1.2-1.4) | **1.2 (1.1-1.4)** |
| Emergency cesarean delivery | 73 860 (8.5) | 547 (0.7) | 1.2 (1.1-1.3) | 1.0 (0.9-1.2) |
| Maternal obesity**  (Body Mass Index ≥ 30) | 24 764 (2.9) | 136 (0.5) | 1.2 (1.0-1.4) | 1.1 (0.9-1.4) |
| Daily smoking early in  pregnancy *** | 46 529 (5.4) | 339 (0.7) | 1.4 (1.3-1.5) | **1.4 (1.3-1.5)** |
| Low education  (High school or less) | 394 578 (45.6) | 2 765 (0.7) | 1.2 (1.2-1.3) | **1.2 (1.2-1.3)** |
| Parental consanguinity | 8 970 (1.0) | 103 (1.1) | 1.9 (1.5-2.3) | **1.9 (1.5-2.3)** |

**†** Data available only for birth cohorts 2008-2014 (N = 391817)
*Adjusted for severe perinatal asphyxia (Apgar 5-min 0-3 and NICU admission) ** Missing data for 77 % of population, not included in analysis. *** Missing data for 15.7 % of population, not included in analysis
